# Heat and fraud: evaluating how room temperature influences fraud likelihood

**DOI:** 10.1186/s41235-020-00261-2

**Published:** 2020-11-19

**Authors:** Huanxu Liu, Jingwen Yang, Yuki Yamada

**Affiliations:** 1 Graduate School of Human-Environment Studies, Kyushu University, 744 Motooka, Nishi-ku, Fukuoka, 819-0395 Japan; 2 Faculty of Arts and Science, Kyushu University, 744 Motooka, Nishi-ku, Fukuoka, 819-0395 Japan

**Keywords:** Fraud, Physical environment, Temperature, Unethical behavior, Cheating, Warmth

## Abstract

Despite the considerable amount of research devoted to understanding fraud, few studies have examined how the physical environment can influence the likelihood of committing fraud. One recent study found a link between room brightness and occurrence of human fraud behaviors. Therefore, the present study aims to investigate how temperature may affect fraud. Based on a power analysis using the effect size observed in a pilot study, we recruited 105 participants and randomly divided them into three temperature groups (warm, medium, and cool). We then counted fraud behaviors in each group and tested for potential significant differences with a Kruskal–Wallis test. Additionally, we used a correlation analysis to determine whether the perceived temperature affected fraud. As a result, regardless of participants’ subjective sensory experience or their physical environment, we did not find that temperature-related factors influence the incidence of fraud. We discussed the potential reason for the results and suggested directions for future research.

## Introduction

The widespread prevalence of fraud has led to numerous studies on its causes and prevention. Such research is interdisciplinary, spanning fields such as physiology, sociology, economy, justice, and education. Psychology has attempted to uncover the mechanism of fraud using personality (Egan and Taylor 2010) and social factors (Gino et al. 2009) as frameworks. An increasingly popular approach is through the lens of environmental psychology. For example, a recent report demonstrated that the brightness of a room can influence fraud likelihood. The hypothesis put forth explaining this phenomenon is that darkness created “illusory anonymity,” causing people to feel safer about committing fraud (Zhong et al. 2010). However, numerous other physical environmental factors could potentially influence fraud behavior but have not been examined. Our study aims to help fill this knowledge gap through investigating the effects of temperature on fraud.

Research in criminology has identified a correlation between crime rates and temperatures worldwide, including countries such as China, Japan, the USA, India, and Australia (e.g., Blakeslee and Fishman 2018; Hu et al. 2017a, b; Hu et al. 2017a, b; Mares 2013; Rotton and Cohn 2003; Schinasi and Hamra 2017; Sommer et al. 2018; Stevens et al. 2019; Takahashi 2017; Tiihonen et al. 2017). Most of these studies examined financial and violent crimes. A separate study also found motives for committing a crime were related to temperature at the scene (Gockel et al. 2014). Taken together, these data suggest that temperature affects behavior on a macroscopic level.

Psychologists began to investigate a potential relationship between temperature and behavior 30 years ago. One study concluded that heat exerts ubiquitous effects on the occurrence of human violence (Anderson, 1989). More recently, Williams and Bargh (2008) reported that physical temperature can alter the closeness of human relationships due to effects on cognition, although the study could not be replicated and its credibility is therefore questioned (Lynott et al. 2014; Chabris et al. 2019). Nevertheless, another study reported that warm conditions result in more pro-social behaviors (Ijzerman and Semin 2009). Further investigations on potential physiological mechanisms revealed that temperature activates both the insular cortex and putamen, thus influencing cognitive function (Sung et al. 2007). A subsequent study also revealed that temperature affected the insula, with consequences for neural activation and behavior (Kang et al. 2011). These findings corroborated a hypothesis (Craig, 2002, 2009), suggesting that the physical environment activates the posterior insula, mid-insula, and anterior insular cortex in that order to generate emotion and cognition. More recently, colder temperatures were found to improve cognitive control capacity (Halali et al. 2017). Based on these studies, there is sufficient evidence to suggest that cognitive modulation by temperature is a factor in human fraud. Following Halali et al. (2017), we developed the following hypothesis.

### Hypothesis (H1)

A low temperature improves cognitive control performance and thus reduces fraud.

Individual perceptions of the same objective temperature may also vary. For example, participants at the same temperature felt colder when asked to recall negative memories (Zhong and Leonardelli 2008). These results indicate that both physical temperature and psychological temperatures (subjective feeling of warmth, called “subjective warmth” below) could affect cognitive control (Halali et al. 2017). Therefore, a deeper understanding of the relationship between temperature and fraud requires the inclusion of subjective warmth. With this in mind, our study aims to test the following hypothesis.

### Hypothesis (H2)

There is a relationship between subjective warmth and fraud occurrence.

To test these hypotheses, we examined previous methods for detecting fraud incidence in the laboratory. One common technique is to create an anonymous environment. Numerous participants are then recruited to take part in a mock test that they themselves score and report. The experimenter can identify fraud occurrence by comparing differences between the question and grading sheets (Gino et al. 2009, 2011; Zhong et al. 2010; Lee et al. 2015). The second method assesses fraudulent behaviors through probability analyses using tools such as coins and dice (e.g., Fischbacher and Heusi 2008; Bryan et al. 2013). For example, a participant may be asked to toss a coin 10 times for a reward dependent on the outcome. Experimenters can then determine the difference in distribution between the reported number and chance (e.g., 50% for coin toss). A recent study modified fraud experiments using dice by digitizing the process (Kocher et al. 2018; Köbis et al. 2019). Through a computer program, participants rolled virtual dice, and their responses were recorded. A final common method involves a confederate (e.g., Niiya et al. 2008; Bocian and Wojciszke, 2014; Bocian et al. 2016). The confederate is the first to cheat when the experimenter leaves the room and invites participants to also cheat. Subsequently, the confederate reports participant decisions to the experimenter.

We considered that an appropriate method for detecting fraud in a laboratory setting should possess three methodological requirements, which is fostering the best environment for increasing the likelihood of fraud; detecting fraud clearly; having few extraneous variables. However, we found that none of the above four methods can meet these three requirements at the same time. Firstly, we cannot determine whether the anonymous environment can foster an appropriate environment for increasing the likelihood of fraud: In most cases, it depends on how anonymous the participants feel they are. Next, the methods which uses tools such as coins and dice cannot detect fraud at the individual level and only can be used when there are enough data to produce a probability similar to chance. Besides, it was pointed out that these methods “do not discriminate between different modes of participant misbehavior” recently (Pascual-Ezama et al. 2020)^1^. Then, we considered that the presence of computers decreases the incidence of fraud because the participants would be aware that their actions can be monitored. Although some studies (e.g., Kocher et al. 2018; Pascual-Ezama et al. 2020) found appropriate rates of fraud by this method, we still would like to find a method making the participants less suspicious and can be used in laboratory more easily. Finally, the confederate itself involves many extraneous variables. For these reasons, we addressed the only issue with the second method, improving its ability to clearly detect fraud. Thus, we ultimately selected this method to test our two hypotheses.

## Methods

### Ethics statement

The experiment was conducted in accordance with guidelines from the Declaration of Helsinki (2013). The ethics committee of Kyushu University approved the protocol (approval number: 2019–003). The experiment was single blind, meaning that participants were given an initial explanation of the experiment, for which they provided informed consent. After participation, researchers disclosed the real purpose of the experiment, and subjects were given another opportunity to provide informed consent. We took steps to protect participant privacy. For more details, see “Procedure.”

### Apparatus and materials

The experiments were performed in a small, dark room of the Psychological Experiment Building at Kyushu University. The room temperatures could be adjusted using the air conditioner. The air conditioner was hung on the wall near the entrance of the laboratory. (Another additional high-power air conditioner was placed in the corner of the room to prevent the situation that only one air conditioner was not enough to adjust the room temperature appropriately.) Because of the place of the air conditioners, the wind from the air conditioners did not directly blow to the participants. The condition “warm” was 29–32 °C, “medium” was 23–26 °C, and “cool” was 17–20 °C. These temperatures were determined based on a pilot study that is described in detail later. We used an independent thermometer to measure the air temperature to determine that the temperature environment was appropriate to the experiment.

To measure subjective warmth, we used a visual analog scale (VAS) to let participants evaluate the degree of how hot (or cool) they felt. On this scale, participants were told that the left end meant that they felt not hot at all and the right end was the hottest. Participants were able to freely mark free a spot on the horizontal line. We used the physical distance between the mark and the left end as an index of subjective warmth.

**Fig. 1:**
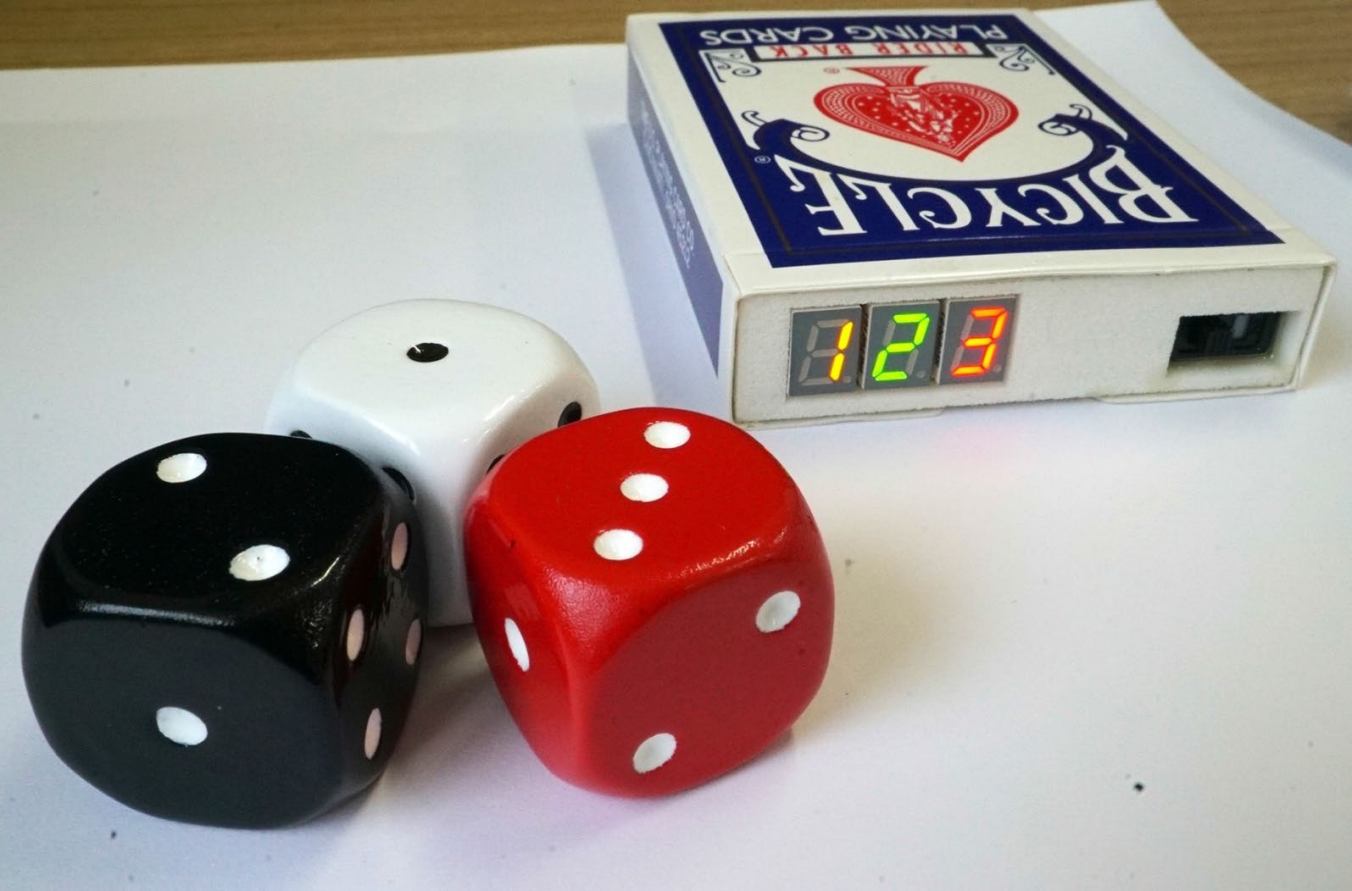
Mental Dice. Numbers on the dice correspond to numbers on the remote controller

To improve fraud measurements, we chose remotely readable dice (Mental Dice, Marc-Antoine) (Fig. 1) instead of normal dice. These dice allow experimenters to know the dice-roll outcomes even while being in a different room. Relatively few people are aware that such an item exists. Nevertheless, we checked for participant knowledge about these dice in a post-experiment interview.^2^

### Procedure

We protected participant privacy by having one experimenter and one recorder. The experimenter recruited participants prior to the experiment. During the experiment, the experimenter interacted with participants and issued identifying numbers, while the recorder used the remote controller to note down participant dice-roll outcomes in a separate room, removing any direct contact with participants. Thus, the experimenter did not know which participants cheated despite having access to personal information. Conversely, the recorder was aware that fraud occurred, but could not link that knowledge to an identity.

Participants were initially informed that they were assisting with research on perceived luckiness in chance-based games. Every participant rolled the dice 20 times. After each roll, the individual wrote down the results and their perceived luckiness on a piece of paper. If the result exceeded 11 (i.e., the expected value of three dice), the participant “won” and received a prize of 150 yen. If the result was ≤ 11, the participant “lost” and 100 yen was subtracted from the accumulated amount of previous trials^3^; in addition, we told participants that if they won less than 8 times, they will get nothing, although we still paid them at least 500 yen after the experiment. Participants were left alone in the laboratory after the experimenter explained the false “luck” test. The recorder then noted down the actual dice-roll results in a separate room. Next, participants were asked to assess subjective warmth using the VAS. Then, the participants were paid and left. After participants left, the experimenter relayed the participant’s ID number and self-reported score to the recorder. If the recorder found a different number between the remote controller and the participant’s self-report, we judged that the participant had committed one act of fraud. We used the frequency of the frauds as the fraud indicator and dependent variable.

### Design

The study used a between-subjects design. Independent variables were temperature (with three levels) and the subjective warmth index (the score of VAS). Participant fraud indicator (the number of times fraud occurred) was the dependent variable.

### Pilot study

The method of the pilot study was almost the same as the main study, with 10 participants in each group. The pilot study provided us with information about the effect size of the temperature factor (*ε*^*2*^), which was 0.0873. This result was used for the power analysis described later. In addition, we found that the data violated the assumption of normality by the Shapiro–Wilk test, *W* = 0.929, *p* = 0.048. Therefore, we decided to use nonparametric tests in the main experiment.

### Data analyses

Between-temperature-group differences in the fraud indicator were analyzed using the Kruskal–Wallis test and the Dwass, Steel, Critchlow–Fligner (DSCF) pairwise comparisons (testing H1). We predicted a significant main effect and a significant difference at least between the warm and cool conditions (i.e., warm > cool). To test H2, we calculated the Spearman’s rank-order correlation between the VAS scores of subjective warmth and the fraud indicator. We predicted a significant positive correlation between them.

### Power analysis and participants

The required sample size was calculated in G*power 3.1.9.3 (By Faul et al. 2009). Under the settings of *ε*^2^ = 0.0873, *α* = 0.05, and power (1 − β) = 0.8 for the Kruskal–Wallis test, the sample size was 105. For a correlation analysis, we set *r* = 0.3 (medium effect; Cohen, 1988), *α* = 0.05, and power (1 − β) = 0.8, resulting in a required sample size of 82. To increase power, we chose n = 105 for our study. We randomly divided 105 participants into three conditions, with 35 participants each. Participants were undergraduate and graduate students at Kyushu University.

### Data exclusion criteria

According to our preregistration, participants would be excluded if they failed to complete all tasks properly or provide adequate data; determined the real purpose of the study before disclosure; knew about Mental Dice; demanded the removal of their data after understanding the real purpose of the study; and those for whom the recorder did not collect their data (e.g., equipment failure).

### Preregistration

The present study is a registered report. The above content was submitted as a first-stage manuscript to this journal (July 01, 2019) and accepted in principle after peer review (December 08, 2019). We then began to collect the data. The following content was written based on data obtained after the preregistration.

## Results

A total of 110 participants (*M*_age_ = 22.4 years, *SD*_age_ = 2.85; 45.71% female, 54.29% male) were recruited to collect the preregistered number of 105 participants while excluding those who violated the exclusion criteria. Based on the exclusion criteria, five participants’ data were excluded from the analyses—four because of equipment failure, and one for detecting the true purpose of the study prior to disclosure. Data from the remaining 105 participants were used as the final dataset for the analysis. Analyses were conducted using jamovi software (2019, Version 1.1.7.0; https://www.jamovi.org/).

### *Preregistered analysis: Between-group differences in the fraud indicator*^4^

**Fig. 2:**
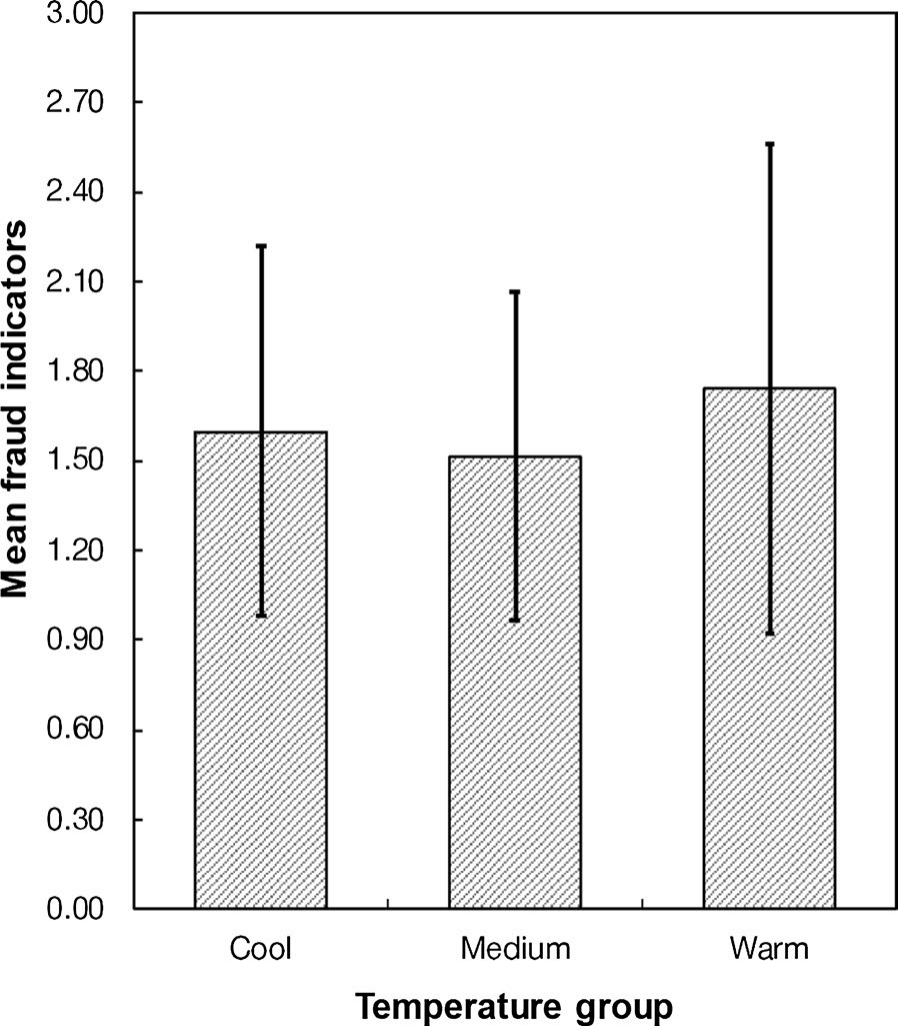
Mean fraud indicators for the three temperature groups. Error bars represent 95% confidence intervals

We summarized the characteristics of fraud for each temperature group (*M*_cool_ = 1.60, *SD*_cool_ = 1.80; *M*_medium_ = 1.51, *SD*_medium_ = 1.60; *M*_warm_ = 1.74, *SD*_warm_ = 2.38). As stated above, we analyzed between-temperature-group differences in the fraud indicator using the Kruskal–Wallis test and Dwass, Steel, Critchlow–Fligner (DSCF) method for pairwise comparisons to test H1. The fraud indicator did not significantly differ between the three groups, *x*^*2*^(2) = 0.0914, *p* = 0.955, ε^*2*^ = 0.001 (Fig. 2). The DSCF pairwise comparisons showed no significant differences between any two groups: cool vs. medium: *W* = − 0.112, *p* = 0.997; cool vs. warm: *W* =  − 0.448, *p* = 0.946; medium vs. warm: *W* = − 0.250, *p* = 0.983.

### Preregistered analysis: a correlation analysis between subjective warmth and the fraud indicator

According to the protocol, we calculated the Spearman’s rank-order correlation coefficients between the VAS scores for subjective warmth and the fraud indicator. We did not find a significant correlation between the two variables, Spearman’s *rho* = − 0.020, *p* = 0.841.

### Unregistered analysis: between-temperature-group differences in subjective warmth

The preregistered analyses showed that the fraud indicator did not significantly differ among the three temperature groups. From these results, we considered the possibility that there was a subjective, rather than physical, temperature effect. That is, our participants may not have experienced a significant difference in subjective temperature among the three conditions, resulting in the null temperature effect. Thus, based on the results of Shapiro–Wilk test, *W* = 0.982, *p* = 0.179, we used the room temperature as an independent variable and the subjective warmth as a dependent variable to carry out a one-way ANOVA and multiple comparisons by Tukey’s method for the analysis of differences in subjective warmth between three temperature groups. The results showed a significant main effect of room temperature, *F* (2, 102) = 39.8, *p* < 0.001. Additionally, the multiple comparisons showed that the subjective warmth was significantly lower in the cool group than in the medium and warm groups and significantly lower in the medium group than in the warm group (cool vs. medium: *M*_Diff_ = − 1.46, *p* = 0.016; cool vs. warm: *M*_Diff_ = − 4.52, *p* < 0.001; medium vs. warm: *M*_Diff_ = − 3.07, *p* < 0.001). The results suggest that even though the participants seemed to be aware of the room temperature, the fraud indicator did not differ significantly between the three groups. Whether temperature differed physically or subjectively, it would still be unable to affect fraud.

### Unregistered analysis: biases of reported numbers

One potential limitation to our methodology is the possibility that any difference between the number on the remote controller and the number reported by the participant could be a calculation error, rather than fraud. If a difference between the participant-reported number and the number on the remote controller was caused by a calculation error, it would not be biased towards positive or negative numbers (because of the random distribution), whereas any difference related to fraud might show a strong bias in favor of positive numbers (linked to more rewards). Thus, we analyzed the difference to check whether it could be attributed to an error or fraud. We extracted the data (participant-reported number—number on the remote controller) and analyzed normality by Shapiro–Wilk test, *W* = 0.785, *p* < 0.001. Next, we performed Wilcoxon signed-rank test between the difference and 0, which was significantly more than 0, z = 3.13, *p* = 0.002, Cohen’s *d* = 0.385. Admittedly, some calculation errors would have occurred inevitably; nonetheless, our results show a distinguishing trait related to fraud, and we concluded that our method was sufficiently sensitive to detect fraud.

## Discussion

### Temperature and fraud

Like room brightness, temperature is an environmental factor affecting many human activities. Previous research has demonstrated that the brightness of a room had a strong effect on fraud (Zhong et al. 2010). However, we could not obtain clear statistical support for an effect of temperature on fraud in the current study. This failure could be attributed to two possible causes, discussed below.

The first possible reason for our results is that the laboratory temperature used in the present study was not appropriate. To provide an appropriate temperature range for the observation of an ideal fraud rate, based on the pilot study (see Additional file 1 for details), we set up three temperature groups: warm (29–32 °C), medium (23–26 °C), and cool (17–20 °C). However, this approach had two limitations. First, temperatures outside the selected temperature range were not examined (less than 17 °C or more than 32 °C). It could be possible that human beings commit fraud under extreme temperatures, as unpleasant stimuli (such as extreme temperatures) have been shown to decrease cognitive resources (Gaoua 2010). Since it might be useful to elucidate the mechanism for determining the commission of fraud, it is worth investigating the issue under more extreme temperatures. Second, we did not examine fraud at 21–22 °C and 27–28 °C, based on Schinasi and Hamra’s (2017) finding that crime rates were highest when the daily heat index was 22.6–28 °C. Similarly, it is possible that the rate of fraud might be highest in a specific temperature range.

The second possible factor contributing to our results is that, compared to room brightness, the effect of temperature on fraud is harder to observe, suggesting that not all human information processing systems are involved in the system determining whether or not to commit fraud. In other words, the visual system (brightness) has a stronger influence on the system determining whether or not to commit fraud than the tactile system (temperature). For further investigation of this issue, future studies should directly compare the effect size of environmental factors related to the functions of different sensory organs (e.g., colors of light, styles of wallpaper, presence of noise, or the hardness of a chair).

The results of the current study did not show a similar effect size as the pilot study (pilot study vs. main experiment: ε^*2*^ = 0.087 vs. ε^*2*^ = 0.001). The first possible cause for this discrepancy is that the sample size of the pilot study was extremely small, and its effect size might therefore be overvalued. Second, the implementation timing differed between the two experiments. The pilot study was conducted in October, and the main experiment in January. According to the records of the Japan Meteorological Agency (https://www.jma.go.jp/jma/indexe.html), the average temperature in Fukuoka, Japan, in October 2019 was 20.5 °C and 9.5 °C in January 2020. In the present study, all participants were wearing their own seasonal clothes. It is undeniable that there may be interactions between the factors investigated in the present study, and those related factors that went unexplored (e.g., outdoor temperature or participants’ clothing).

### Constraints on generalizability

Although all participants in the present study were students aged 18 to 33 years, we do not consider the results to be restricted to students. Nevertheless, we conjectured that some participant factors might have affected the results. The first is moral sense, as human morality is directly involved in fraud. In order to reproduce results from the current study, it is necessary for participants to have a moral sense similar to university students. Moral sense might be further influenced by factors like experience and gender (Boccia et al. 2017). The second factor is participants’ economic situation—a big or important reward may motivate participants to commit fraud. In other words, our results may be reproduced when participants’ economic situation approximates that of general university students. The cost of living, including tuition, for the participant, a national university student, is around 127,000 yen per month (https://www.jasso.go.jp/about/statistics/gakusei_chosa/index.html), which is about the equal amount as the latest top-of-the-line smartphone.

The stimulus used in the present study was temperature. Since temperature is one of the natural environmental factors, it has few restrictions. However, Gaoua (2010) reported that, compared to normal environments, human behavior might be different under unpleasant environmental conditions, which can decrease cognitive resources. Since the temperature stimuli used in the present study are experienced by participants in their daily lives, we suggest adjusting the stimulus (the temperature range) according to the temperature range of the subjects’ milieu in order to replicate the current results. In other words, if the main experiment was carried out after a pilot study that investigated the temperature range of the location, the results from a study of the effects of temperature might be more accurate.

There are additional points to consider in terms of procedure that could cause future studies to fail to reproduce the results, or could produce a fraud rate different from that seen in the present study. The first point to consider is the setup of a laboratory. Zhong et al. (2010) found that a dark environment could increase fraudulent behavior; therefore, in the current study, we set up the laboratory to be as dark as possible. (The lighting did not hinder the rolling of the dice and the recording of results.) Further, electronic appliances such as personal computers might give participants the sense that they are “under surveillance”; for this reason, we merely placed a pen, the recording paper, and the dice in participants’ range of vision. The second point to consider is the characteristics of the experimenters. The experimenters in the present study were female college students. Participants may feel susceptible to more aggressive behavior in the case of male experimenters than female experimenters, evoking stronger fear (Harris and Miller 2000). In such situations, the fraud rate may also be lower. The reward system is the third consideration. Our maximum reward was 3000 yen—a relatively high reward compared to other psychological experiments. The incidence of fraud will fluctuate significantly based on the reward amount, and we told the participants if they won fewer than eight times, the reward would become 0. It is possible that the participants committed more fraud to avoid losing their reward. Future studies should use a similar reward system to increase participants’ motivation to commit fraud. The final consideration is the experiment duration. To avoid calculation errors, we did not limit the duration of the experiment in the present study. While it is not clear yet whether time-related pressure could influence the incidence of fraud (Shalvi et al. 2012; Van der Cruyssen et al. 2020), we suggest that future studies should not limit the experiment duration either, in order to reproduce the results of the present study. In sum, future study procedures should provide an ideal environment to increase the likelihood of fraud by using non-aggressive experimenters, a relatively high reward system, and no limit on experiment duration.

We have no reason to believe that the results would be influenced by other participant characteristics, materials, or contextual considerations (Simons et al. 2017).

### The utility of the method used in the present study

To ensure that the method used in the present study was appropriate for detecting fraud in a laboratory setting, we reviewed the three methodological requirements of available fraud-detection experiments. The first requirement is to foster the best environment for increasing the likelihood of fraud. The respective rates of fraud (the number of participants who committed fraud/the number of participants in the group) for each temperature group were 65.71% (cool), 62.86% (medium), and 60.00% (warm). All three groups showed medium-ranked fraud rates; an excessive or insufficient fraud rate would mean that the data could not be analyzed. Our results suggest that we had created an ideal environment for increasing the likelihood of fraud for all three groups. The second requirement is a clear method for detecting fraud. Since all outcomes were known via the remote controller, we regarded our method as being able to detect fraud more clearly than traditional methods. As previously mentioned, calculation errors might pose a problem in our method; in fact, calculation errors are unavoidable. Thus, to decrease the likelihood of calculation errors, we gave participants the following instruction: “This experiment has no time limit. Please be careful to ensure the results are accurate.” Additionally, we certified that fraudulent activities were detected clearly through our earlier explorative analysis. Finally, the presence of fewer extraneous variables than traditional methods is the last requirement. The method used in the present study had fewer extraneous variables than traditional methods due to using neither computers nor confederates. As mentioned above, the presence of computers or other electronic devices in the vicinity during an experiment that examines cheating behavior, such as dummy security cameras on the street, may decrease the incidence of fraud. The present method provides a simple, direct means to research fraud, and it is expected to ring in a new paradigm for fraud-detection experiments.

### Fraud rate per outcome

**Fig. 3:**
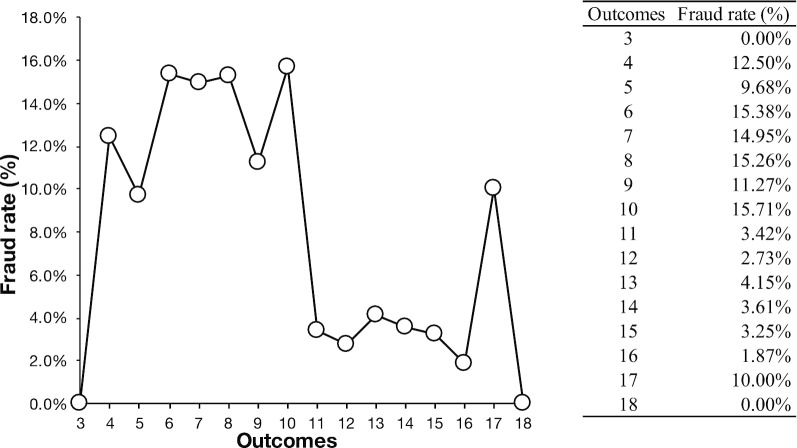
The fraud rate for each result

The conditions that enable fraudulent behavior can be investigated by using existing data, and we were interested to know whether the outcomes affected the fraud rate. If participants were more likely to commit one act of fraud when they were likely to be a winner, the fraud rate would be higher than other rolls when the outcomes of the roll were 10. (When the outcomes of the roll were 11, participants could win the reward. See “Footnote 3” for more details.) In order to explore this, we calculated the rate of fraud for each of the possible outcomes (Fig. 3). Figure 3 seems to suggest that the outcome “10” had the highest fraud rate (15.71%); however, this is minimally different from the outcome for “8” (15.26%) or “6” (15.38%). We also found that when the participants obtained the losing outcomes from “4” to “10,” their fraud rate was about 10– 15%. However, when the outcome was 3, the fraud rate was 0% (0 times / 10 times). We could not conclude whether it is because the participants considered the outcome 3 was too bad to lie, or the participants considered the result of 1-1-1 has some special meaning (a different kind of luck). These issues could be examined more thoroughly in future studies.

## Conclusion

Regardless of participants’ subjective sensory experience or their objective environment, the present study did not find that temperature-related factors influence the incidence of fraud. We discussed the possible cause of the results and suggested directions for future research to elucidate the relationship between temperature and fraud. The method used in the present study presents a new paradigm for fraud-detection experiments.

## Supplementary information

**Additional file 1.** A supplemental description of the pilot studies.

## Data Availability

The datasets are available at Open Science Framework (10.17605/OSF.IO/PR475).

## Footnotes

1 As some new related papers have been published after we completed the Stage 1 manuscript, we have revised some related descriptions in the Stage 1 manuscript. Such modifications do not change the initial logic or structure of Stage 1. Nor do they affect the hypotheses or conclusions of this study at all.

2 Because the participants who finished the experiment might tell the purpose of the experiment to other potential participants, we did not ask participants if they knew about the Mental Dice immediately after the experiment. Then, after completing all the experiments, we sent a unified email about the information disclosure of the experiment to the participants. There was no report about knowing the Mental Dice.

3 We also made an error of expression in the Stage 1 manuscript. The preregistered plan specified “If the result exceeded 11 (i.e., the expected value of three dice), the participant ‘won’ and received a reward of 150 yen.” However, the expected value of the three dice is 10.5. Thus, when the result was 11, we admitted that the participant had “won,” and received a reward of 150 yen.

4 According to the protocol, we considered a participant to have committed one act of fraud if there was a difference in the number reported by the remote controller and participants’ self-reported number. However, we encountered another situation that we consider to be fraudulent behavior, namely the “re-roll,” where participants rolled the dice again when they regarded their result to be inappropriate. As this behavior provided a chance for increased rewards–similar to declaring a false result–we decided that a “re-roll” would also constitute an act of fraud, regardless of participants’ self-reported results. The re-roll is considered as a new measure of dishonest behavior in a recent study (Pascual-Ezama et al. 2020).

## Supplementary information

A supplemental description of our pilot studies is available for this paper at 10.1186/s41235-020-00261-2.

## References

[CR1] Anderson CA (1989). Temperature and aggression: ubiquitous effects of heat on occurrence of human violence. Psychological Bulletin.

[CR2] Blakeslee DS, Fishman R (2018). Weather shocks, agriculture, and crime evidence from India. Journal of Human Resources.

[CR3] Boccia M, Verde P, Angelino G, Carrozzo P, Vecchi D, Piccardi L, Colangeli S, Cordellieri P, Ferlazzo F, Giannini AM (2017). Effect of professional expertise and exposure to everyday life decision-making on moral choices. Neuroscience Letters.

[CR4] Bocian K, Baryla W, Wojciszke B (2016). When dishonesty leads to trust: Moral judgments biased by self-interest are truly believed. Polish Psychological Bulletin.

[CR5] Bocian K, Wojciszke B (2014). Self-interest bias in moral judgments of others’ actions. Personality and Social Psychology Bulletin.

[CR6] Bryan CJ, Adams GS, Monin B (2013). When cheating would make you a cheater: Implicating the self prevents unethical behavior. Journal of Experimental Psychology: General.

[CR7] Chabris CF, Heck PR, Mandart J, Benjamin DJ, Simons DJ (2019). No evidence that experiencing physical warmth promotes interpersonal warmth: Two failures to replicate Williams and Bargh (2008). Social Psychology.

[CR8] Cohen J (1988). Statistical Power Analysis for the Behavioral Sciences.

[CR9] Craig AD (2002). How do you feel? Interoception: The sense of the physiological condition of the body. Nature Reviews Neuroscience.

[CR10] Craig AD (2009). How do you feel–now? The anterior insula and human awareness. Nature Reviews Neuroscience.

[CR11] Egan V, Taylor D (2010). Shoplifting, unethical consumer behaviour, and personality. Personality and Individual Differences.

[CR12] Fischbacher U, Föllmi-Heusi F (2008). Lies in disguise—An experimental study on cheating. Journal of the European Economic Association.

[CR13] Gaoua N (2010). Cognitive function in hot environments: A question of methodology. Scandinavian Journal of Medicine & Science in Sports.

[CR14] Gino F, Ayal S, Ariely D (2009). Contagion and differentiation in unethical behavior: The effect of one bad apple on the barrel. Psychological Science.

[CR15] Gino F, Schweitzer ME, Mead NL, Ariely D (2011). Unable to resist temptation: How self-control depletion promotes unethical behavior. Organizational Behavior and Human Decision Processes.

[CR16] Gockel C, Kolb PM, Werth L (2014). Murder or not? Cold temperature makes criminals appear to be cold-blooded and warm temperature to be hot-headed. PLoS ONE.

[CR17] Halali E, Meiran N, Shalev I (2017). Keep it cool: Temperature priming effect on cognitive control. Psychological Research Psychologische Forschung.

[CR18] Harris MB, Miller KC (2000). Gender and perceptions of danger. Sex Roles: A Journal of Research.

[CR19] Hu X, Chen P, Huang H, Sun T, Li D (2017). Contrasting impacts of heat stress on violent and nonviolent robbery in Beijing China. Natural Hazards.

[CR20] Hu X, Wu J, Chen P, Sun T, Li D (2017). Impact of climate variability and change on crime rates in Tangshan, China. Science of the Total Environment.

[CR21] IJzerman, H., & Semin, G. R. (2009). The thermometer of social relations: Mapping social proximity on temperature. Psychological Science.

[CR22] Kang Y, Williams LE, Clark MS, Gray JR, Bargh JA (2011). Physical temperature effects on trust behavior: the role of insula. Social Cognitive and Affective Neuroscience.

[CR23] Köbis N, van der Lingen S, Cruz TDD, Iragorri-Carter D, van Prooijen JW, Righetti F, Van Lange PA (2019). The look over your shoulder: Unethical behaviour decreases in the physical presence of observers. PsyArXiv.

[CR24] Kocher MG, Schudy S, Spantig L (2018). I lie? We lie! Why? Experimental evidence on a dishonesty shift in groups. Management Science.

[CR25] Lee JJ, Gino F, Jin ES, Rice LK, Josephs RA (2015). Hormones and ethics: Understanding the biological basis of unethical conduct. Journal of Experimental Psychology: General.

[CR26] Lynott D, Corker K, Wortman J, Connell L, Donnellan MB, Lucas R (2014). Replication of “experiencing physical warmth promotes interpersonal warmth” by Williams & Bargh (2008). Social Psychology.

[CR27] Mares D (2013). Climate change and crime: monthly temperature and precipitation anomalies and crime rates in St. Louis, MO 1990–2009. Crime, Law and Social Change.

[CR28] Niiya Y, Ballantyne R, North MS, Crocker J (2008). Gender, contingencies of self-worth, and achievement goals as predictors of academic cheating in a controlled laboratory setting. Basic and Applied Social Psychology.

[CR30] Pascual-Ezama, D., Prelec, D., Muñoz, A., & Gil-Gómez de Liaño, B. (2020). Cheaters, liars, or both? A new classification of dishonesty profiles. *Psychological Science*. Advance online publication.10.1177/0956797620929634. 10.1177/0956797620929634 32780626

[CR31] Rotton J, Cohn EG (2003). Global warming and US crime rates: An application of routine activity theory. Environment and Behavior.

[CR32] Schinasi LH, Hamra GB (2017). A time series analysis of associations between daily temperature and crime events in Philadelphia Pennsylvania. Journal of Urban Health.

[CR33] Shalvi S, Eldar O, Bereby-Meyer Y (2012). Honesty requires time (and lack of justifications). Psychological Science.

[CR34] Simons DJ, Shoda Y, Lindsay DS (2017). Constraints on generality (COG): A proposed addition to all empirical papers. Perspectives on Psychological Science.

[CR35] Sommer AJ, Lee M, Bind MAC (2018). Comparing apples to apples: An environmental criminology analysis of the effects of heat and rain on violent crimes in Boston. Palgrave Communications.

[CR36] Stevens HR, Beggs PJ, Graham PL, Chang HC (2019). Hot and bothered? Associations between temperature and crime in Australia. International Journal of Biometeorology.

[CR37] Sung EJ, Yoo SS, Yoon HW, Oh SS, Han Y, Park HW (2007). Brain activation related to affective dimension during thermal stimulation in humans: A functional magnetic resonance imaging study. International Journal of Neuroscience.

[CR38] Takahashi R (2017). Climate, crime, and suicide: Empirical evidence from Japan. Climate Change Economics.

[CR39] The jamovi project (2019). *jamovi.* (Version 1.0) [Computer Software]. Retrieved from https://www.jamovi.org.

[CR40] Tiihonen J, Halonen P, Tiihonen L, Kautiainen H, Storvik M, Callaway J (2017). The association of ambient temperature and violent crime. Scientific Reports.

[CR41] Van der Cruyssen I, D’hondt J, Meijer E, Verschuere B (2020). Does honesty require time? Two preregistered direct replications of Experiment 2 of Shalvi, Eldar, and Bereby-Meyer (2012). Psychological Science.

[CR42] Williams LE, Bargh JA (2008). Experiencing physical warmth promotes interpersonal warmth. Science.

[CR43] World Medical Association (2013). Declaration of Helsinki: Ethical principles for medical research involving human subjects. JAMA.

[CR44] Zhong CB, Bohns VK, Gino F (2010). Good lamps are the best police: Darkness increases dishonesty and self-interested behavior. Psychological Science.

[CR45] Zhong CB, Leonardelli GJ (2008). Cold and lonely: Does social exclusion literally feel cold?. Psychological Science.

