# Supplementary material for: Heat and fraud: evaluating how room temperature influences fraud likelihood
Source: Cogn Res Princ Implic. 2020 Nov 19;5:60. doi: 10.1186/s41235-020-00261-2 (PMC7677414; doi:10.1186/s41235-020-00261-2)
Supplement: Supplementary file 1 — Additional file 1. A supplemental description of the pilot studies. [file 41235_2020_261_MOESM1_ESM.pdf]

Supplementary material

Heat and fraud: Evaluating how room temperature influences fraud likelihood

Huanxu Liu, Jingwen Yang, and Yuki Yamada

A supplemental description of our pilot studies

We carried out two pilot studies to estimate the most accurate effect size possible for the power analysis because we found an issue in the first pilot study. A supplemental description for our pilot studies is provided in this section.

Pilot Study 1

Method

The method of Pilot Study 1 was almost the same as that of the main study, except for the temperature range and the reward system. In Pilot Study 1, the temperature range in the warm condition was 22–24°C, 19–21°C in the medium condition, and 15–18°C in the cool condition. The warm and cool conditions were set based on the findings of an earlier study (IJzerman & Semin, 2009). Moreover, we set the medium condition to be the average value of the warm and cool conditions. In addition, we told our participants that they would gain at least 500 yen.

However, we stopped this experiment at nine participants as there was a problem with the method that could lead to a very low incidence of fraud. We postulated that the reason for the low incidence of fraud might be that the guaranteed smallest reward was 500 yen, implying that participants could receive a certain amount of money without much effort, which might reduce the motivation for fraud. Thus, in Pilot Study 2

we changed the reward system and told participants that if they won fewer than eight times, they would gain nothing (although we still paid them at least 500 yen after the experiment).

Results

Since we stopped Pilot Study 1, we did not analyze any data generated from it. As mentioned above, the fraud rate was zero for almost all participants.

Pilot Study 2

Method

As we could not calculate the effect size in Pilot Study 1, we carried out a second pilot study. Except for the reward system, which we had revised from the first pilot study, the low incidence of fraud in Pilot Study 1 was further attributed to the temperature setting in all conditions being similarly mild. Therefore, we changed the temperature conditions from warm: 22–24°C, medium: 19–21°C, and cool: 15–18°C, to warm: 29–32°C, medium: 23–26°C, and cool: 17–20°C. The method of Pilot Study 2 was the same as for the main study, with 10 participants in each group (i.e., 30 participants in total).

Results

We found that the data violated the assumption of normality by the Shapiro–Wilk test, $W = .929$, $p = .048$. The median frequencies of fraud in each temperature condition were 0.5 (cool), 1.0 (medium), and 2.5 (warm). Pilot Study 2 provided us with information about the effect size of the temperature factor, $\varepsilon^2 = 0.0873$.

References

IJzerman, H., & Semin, G. R. (2009). The thermometer of social relations: Mapping social proximity on temperature. *Psychological Science*, *20*(10), 1214–1220.
